# Supplementary material for: Stapling of Peptides Potentiates the Antibiotic Treatment of Acinetobacter baumannii In Vivo
Source: Antibiotics (Basel). 2022 Feb 19;11(2):273. doi: 10.3390/antibiotics11020273 (PMC8868297; doi:10.3390/antibiotics11020273)
Supplement: Supplementary file 1 [file antibiotics-11-00273-s001.zip › antibiotics-1591224-supplementary.pdf]

Supplementary documents to Stapling of Peptides Potentiates: the Antibiotic Treatment of *Acinetobacter baumannii* In Vivo

Gina K. Schouten [1,†], Felix M. Paulussen [2,3,†], Oscar P. Kuipers [4], Wilbert Bitter [1,3,5], Tom N. Grossmann [2,3,*] and Peter van Ulsen [3,5,*]

[1] Medical Microbiology and Infection Control (MMI), Amsterdam UMC Location Vumc, De Boelelaan 1108, 1081 HZ Amsterdam, The Netherlands; g.schouten@amsterdamumc.nl (G.K.S.); w.bitter@amsterdamumc.nl (W.B.)
[2] Department of Chemistry and Pharmaceutical Sciences, Vrije Universiteit Amsterdam, De Boelelaan 1085, 1081 HV Amsterdam, The Netherlands; f.m.paulussen@vu.nl (F.M.P.); t.n.grossmann@vu.nl (T.N.G.)
[3] Amsterdam Institute of Molecular and Life Sciences (AIMMS), Vrije Universiteit Amsterdam, De Boelelaan 1085, 1081 HV Amsterdam, The Netherlands; f.m.paulussen@vu.nl (F.M.P.); w.bitter@amsterdamumc.nl (W.B.); t.n.grossmann@vu.nl (T.N.G.); j.p.van.ulsen@vu.nl (P.v.U.)
[4] Department of Molecular Genetics, Groningen Biomolecular Sciences and Biotechnology Institute, University of Groningen, Nijenborgh 7, 9747 AG Groningen, The Netherlands; o.p.kuipers@rug.nl (O.P.K.)
[5] Department of Molecular Microbiology, Vrije Universiteit Amsterdam, De Boelelaan 1085, 1081 HV Amsterdam, The Netherlands; w.bitter@amsterdamumc.nl (W.B.); j.p.van.ulsen@vu.nl (P.v.U.)
* Correspondence: t.n.grossmann@vu.nl (T.N.G.); j.p.van.ulsen@vu.nl (P.v.U.)
† These authors contributed equally to this work.

Table of Contents

Supplementary figures **3**

Peptide analytics 3

Supplementary figure S1. Sequences and analytical details of peptide L8 and stapled variants 3

Toxicity on zebrafish larvae and haemolytic activity of linear and stapled L6 and L8 11

Supplementary figure S2. Survival rates of zebrafish larvae injected with stapled variants of L6 and L8 and vancomycin 11

Supplementary figure S3. Haemolysis of peptide-treated red blood cells 12

Peptide design 13

Supplementary figure S4. Design and predicted peptide structures 13

Supplementary tables **14**

Peptide analytics 14

Supplementary table S1. Sequences and analytical details of peptide L6/L8 and stapled variants 14

In vitro activity of linear and stapled L6 and L8 against Gram-negative bacteria 15

Supplementary table S2. *In vitro* antimicrobial activities of L6 and vancomycin against Gram-negative clinical isolates 15

Supplementary table S3. *In vitro* antimicrobial activities of L8 and vancomycin against Gram-negative clinical isolates 16

Supplementary table S4. *In vitro* antimicrobial activity of vancomycin, L6 or L8 against *A. baumannii* in presence of lipopolysaccharide 17

Supplementary table S5. *In vitro* antimicrobial activities of stapled variants of L6 or L8 and vancomycin against *A. baumannii* 18

Toxicity of linear and stapled L6 and L8 on Zebrafish larvae 19

Supplementary table S6. Survival percentages of zebrafish larvae injected with stapled variants of L6 or L8 or combinations of the stapled peptides and vancomycin 19

In vivo activity of linear and stapled L6 and L8 against *Acinetobacter baumannii* 20

Supplementary table S7. Survival percentages of *A. baumannii* infected zebrafish larvae treated with combinations of vancomycin and stapled variants of L6 or vancomycin and stapled variants of L8....................20

Supplementary table S8. Survival percentages of *A. baumannii* infected zebrafish larvae treated with combinations of rifampicin or erythromycin and L8S1....................21

Supplementary figures

Peptide analytics

Peptide	Sequence	m/z calc.	m/z found
L6	RRLFRRILRWL	792.5	792.6 $[M+2H]^{2+}$

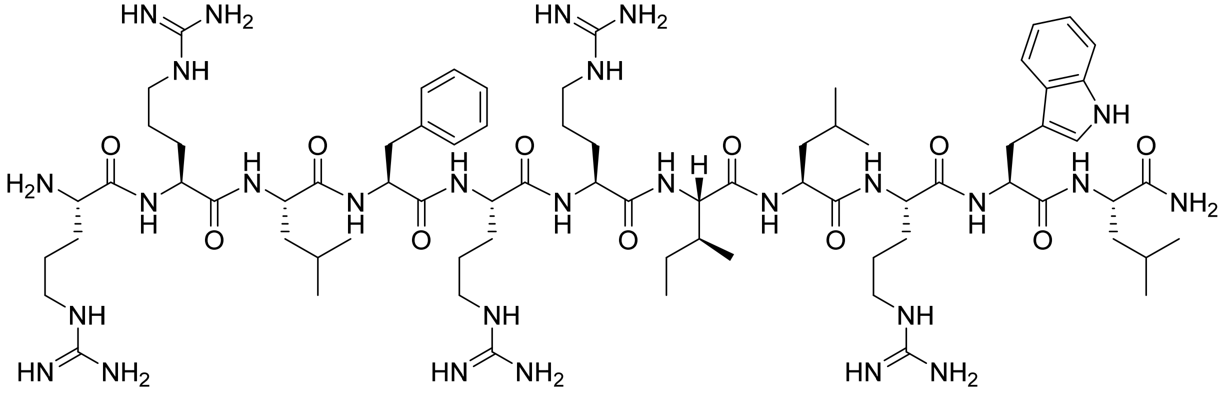

Chemical Formula: $C_{74}H_{126}N_{28}O_{11}$
Molecular Weight: 1584.01

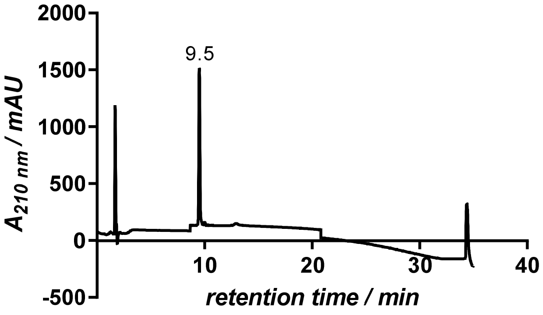

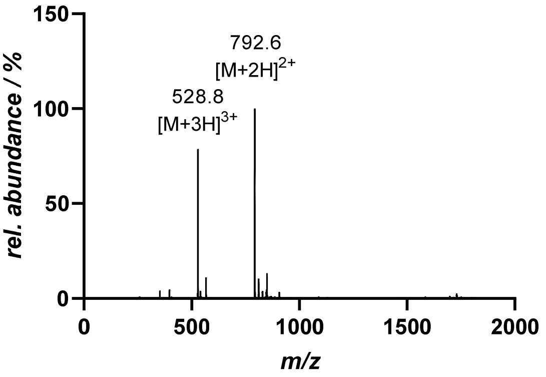

Peptide	Sequence	m/z calc.	m/z found
L6S1	RR(S5)FRR(S5)LRWL	804.0	804.7 $[M+2H]^{2+}$

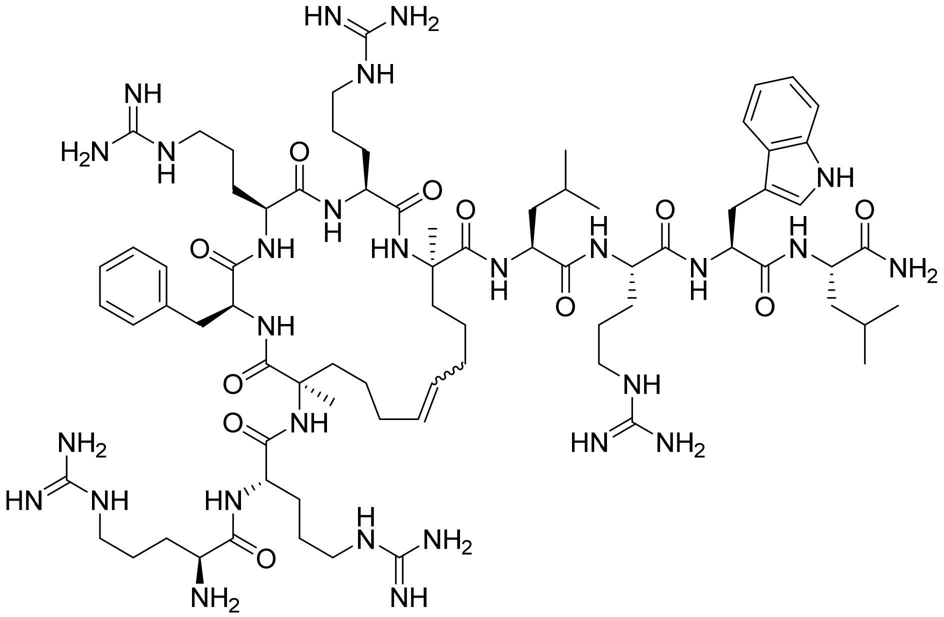

Chemical Formula: $C_{76}H_{126}N_{28}O_{11}$
Molecular Weight: 1608,03

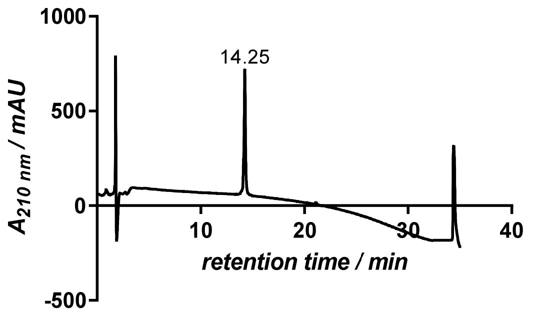

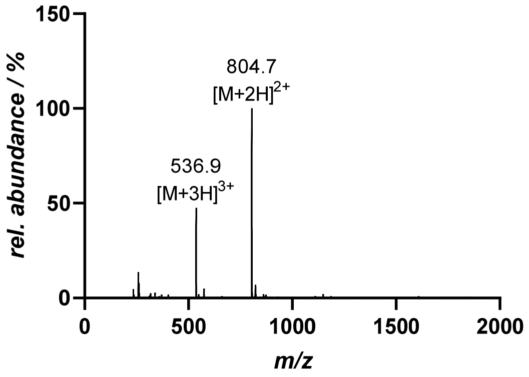

Peptide	Sequence	m/z calc.	m/z found
L6S2	RRLFRR(S5)LRW(S5)	804.0	804.7 $[M+2H]^{2+}$

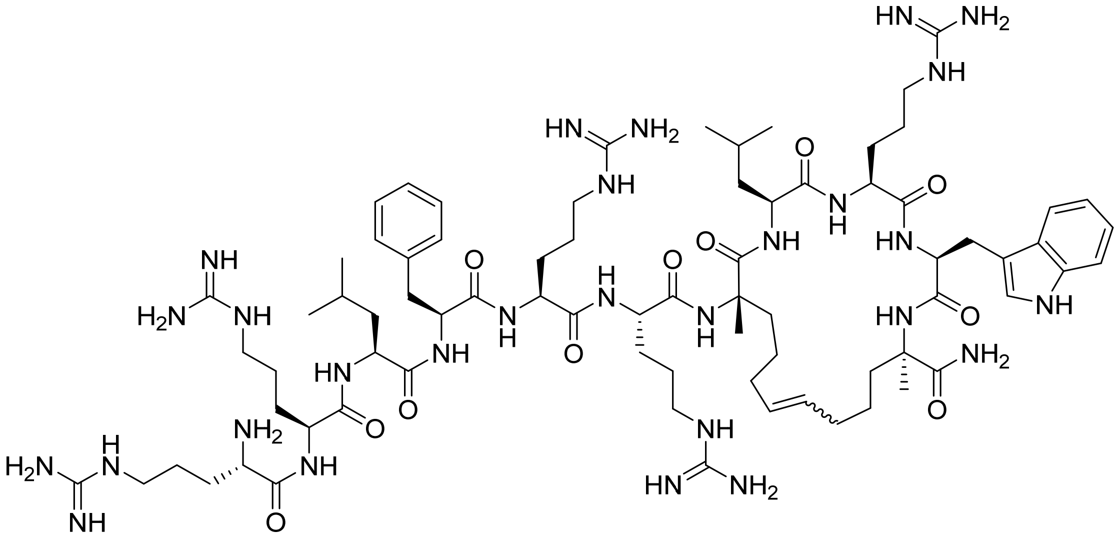

Chemical Formula: $C_{76}H_{126}N_{28}O_{11}$
Molecular Weight: 1608,03

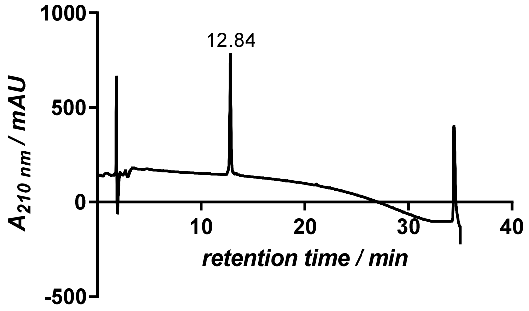

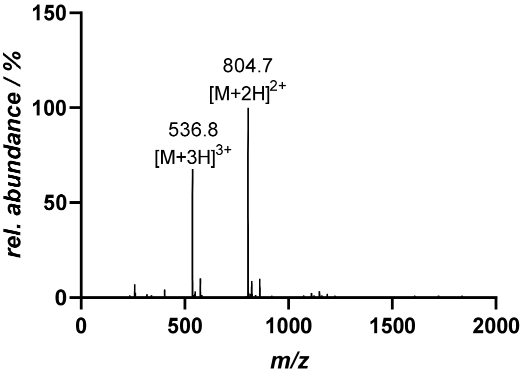

Peptide	Sequence	m/z calc.	m/z found
L6S3	RRL(R8)RRILRW(S5)	809.1	808.7$[M+2H]^{2+}$

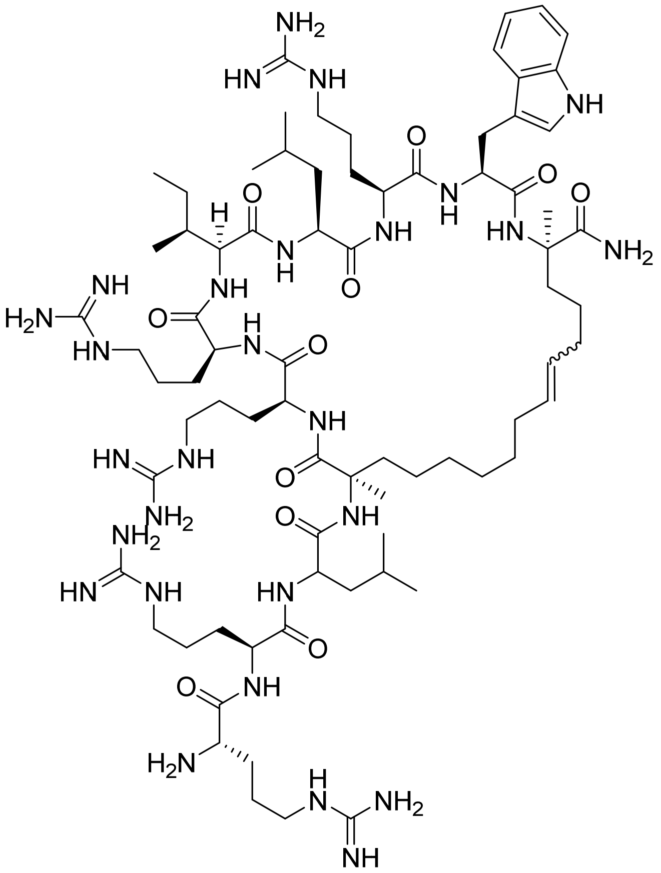

Chemical Formula: $C_{76}H_{134}N_{28}O_{11}$
Molecular Weight: 1616,09

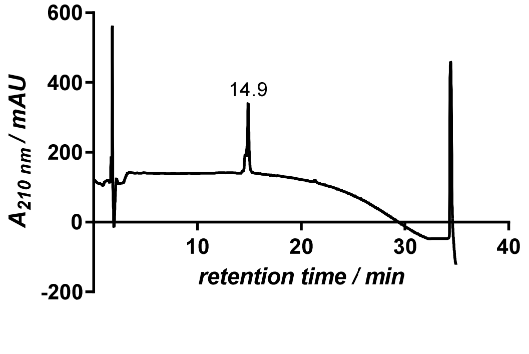

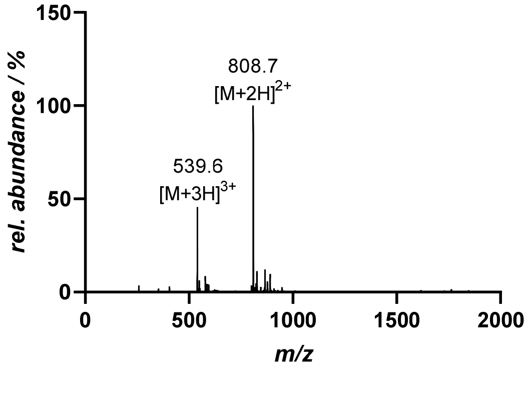

Peptide	Sequence	m/z calc.	m/z found
L8	KRIVQRIKKWLR	812.0	812.3 $[M+2H]^{2+}$

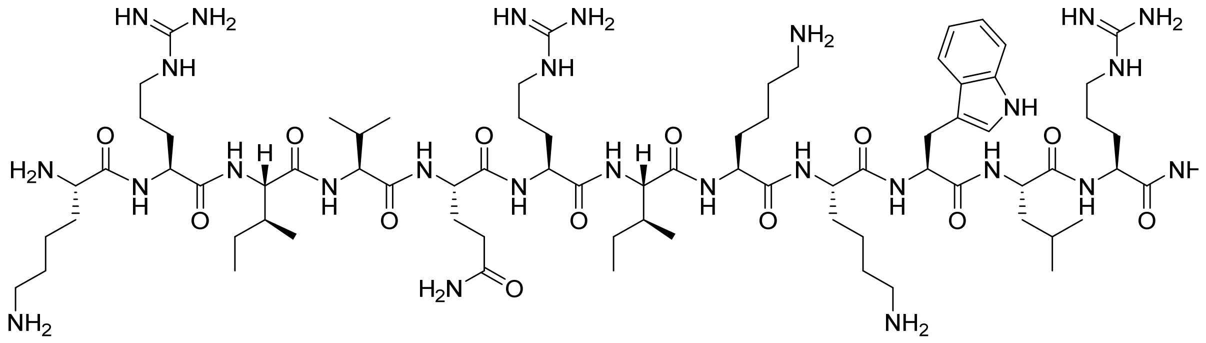

Chemical Formula: $C_{75}H_{135}N_{27}O_{13}$
Molecular Weight: 1623,08

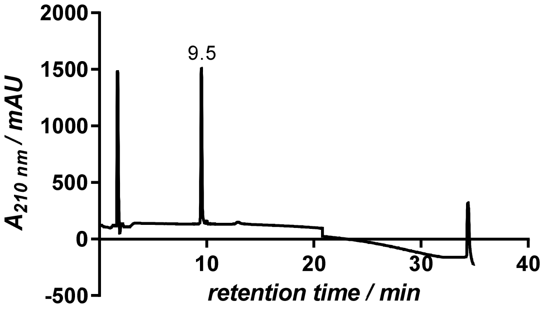

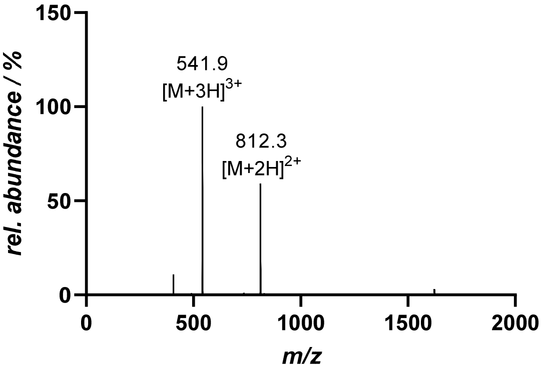

Peptide	Sequence	m/z calc.	m/z found
L8S1	KR(S5)VQR(S5)KKWLR	824.0	824.1 $[M+2H]^{2+}$

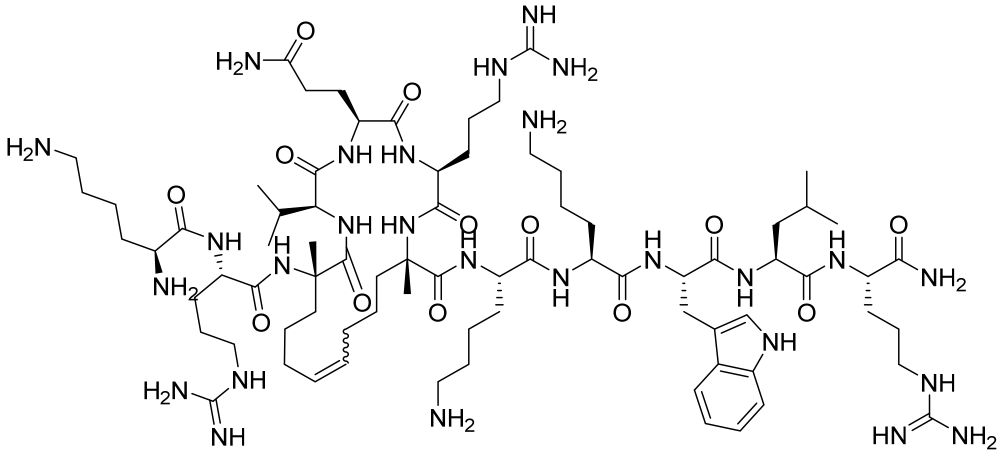

Chemical Formula: $C_{77}H_{135}N_{27}O_{13}$
Molecular Weight: 1647,10

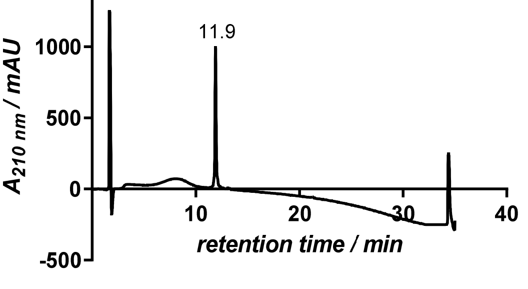

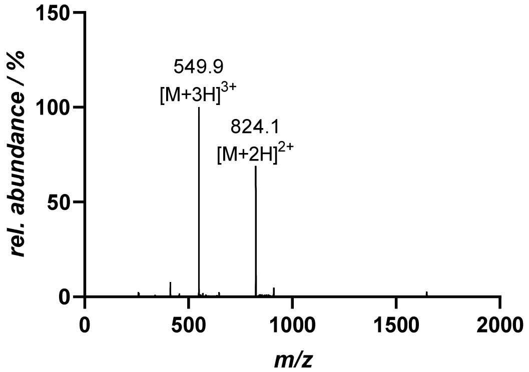

Peptide	Sequence	m/z calc.	m/z found
L8S2	KRIVQR(S5)KKW(S5)R	824.0	824.1 [M+2H]2+

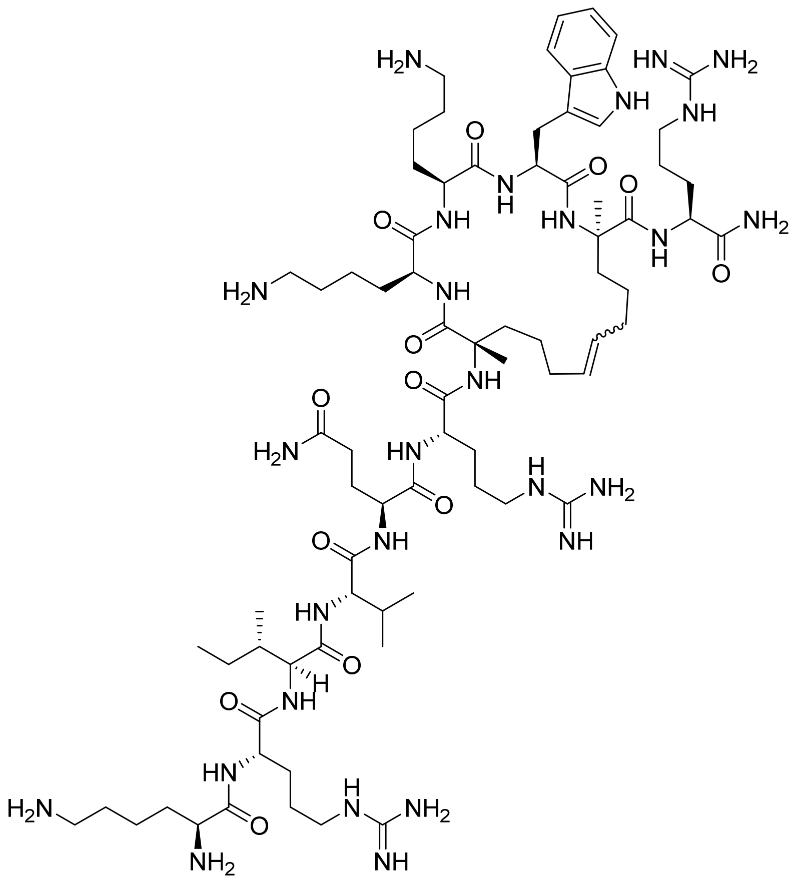

Chemical Formula: $C_{77}H_{135}N_{27}O_{13}$
Molecular Weight: 1647,10

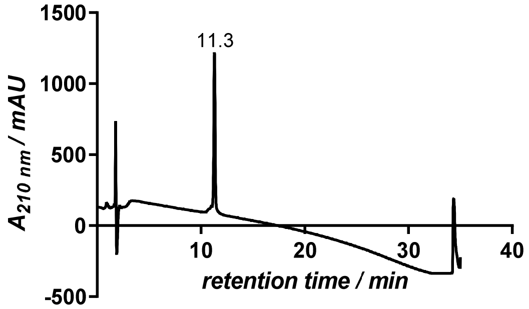

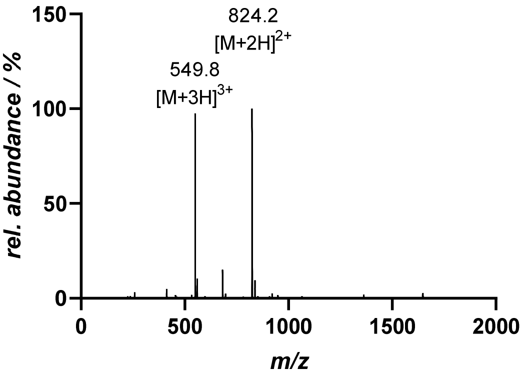

Peptide	Sequence	m/z calc.	m/z found
L8S3	KRI(R8)QRIKKW(S5)R	852.1	852.2 $[M+2H]^{2+}$

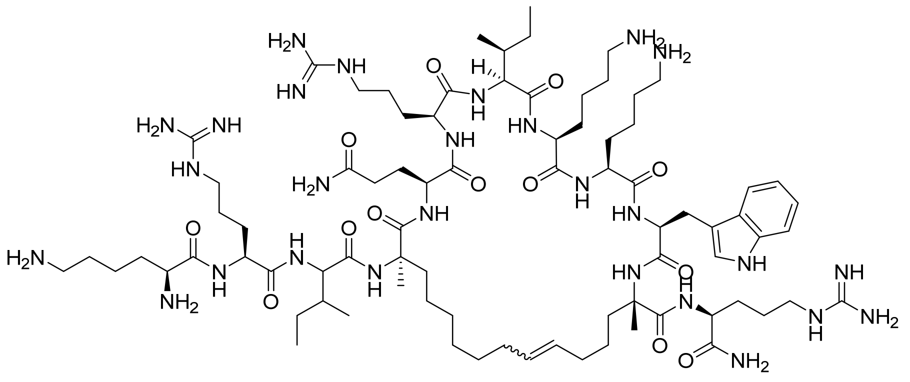

Chemical Formula: $C_{81}H_{143}N_{27}O_{13}$
Molecular Weight: 1703,21

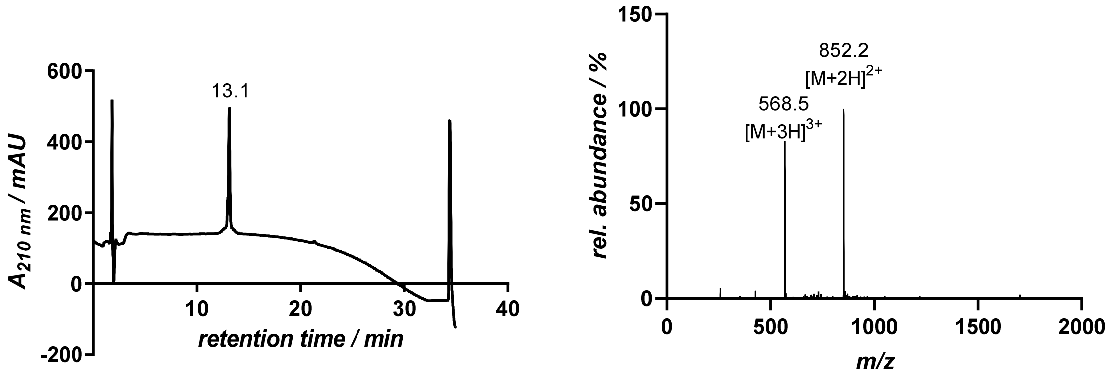

Supplementary figure S1. Sequences and analytical details of peptide L8 and stapled variants. *Purity and mass-to-charge ratio was determined via analytical RP-HPLC-MS with absorption at 210 nm. S5: (S)-N-Fmoc-2-(4′-pentenyl)alanine, R8: (R)-N-Fmoc-2-(7′-octenyl)alanine. HPLC chromatograms: 5-95 % solvent B (MeCN + 0.1% FA + 0.01% TFA), (H_2O + 0.1% FA + 0.01% TFA) in 30 min and MS spectrum (positive mode).*

Toxicity on zebrafish larvae and haemolytic activity of linear and stapled L6 and L8

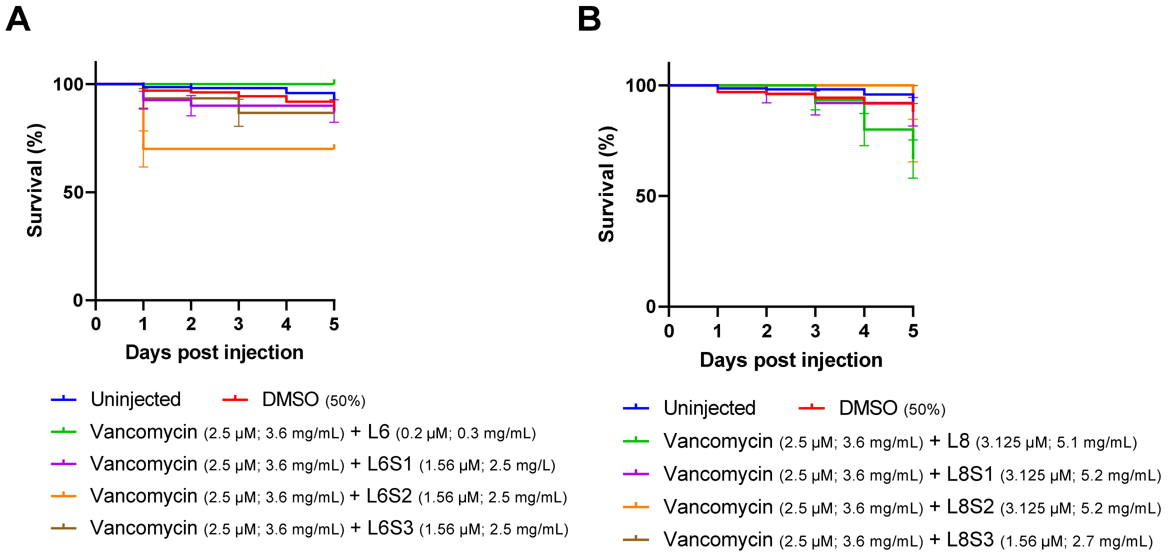

Supplementary figure S2. Survival rates of zebrafish larvae injected with stapled variants of L6 and L8 and vancomycin*. Zebrafish larvae were injected with combinations of stapled variants of L6 and vancomycin (A) or combinations of stapled variants of L8 and vancomycin (B). Surviving larvae were identified by heartbeat over a period of five days post injection. The data are presented as mean ± standard deviation from three independent experiments.*

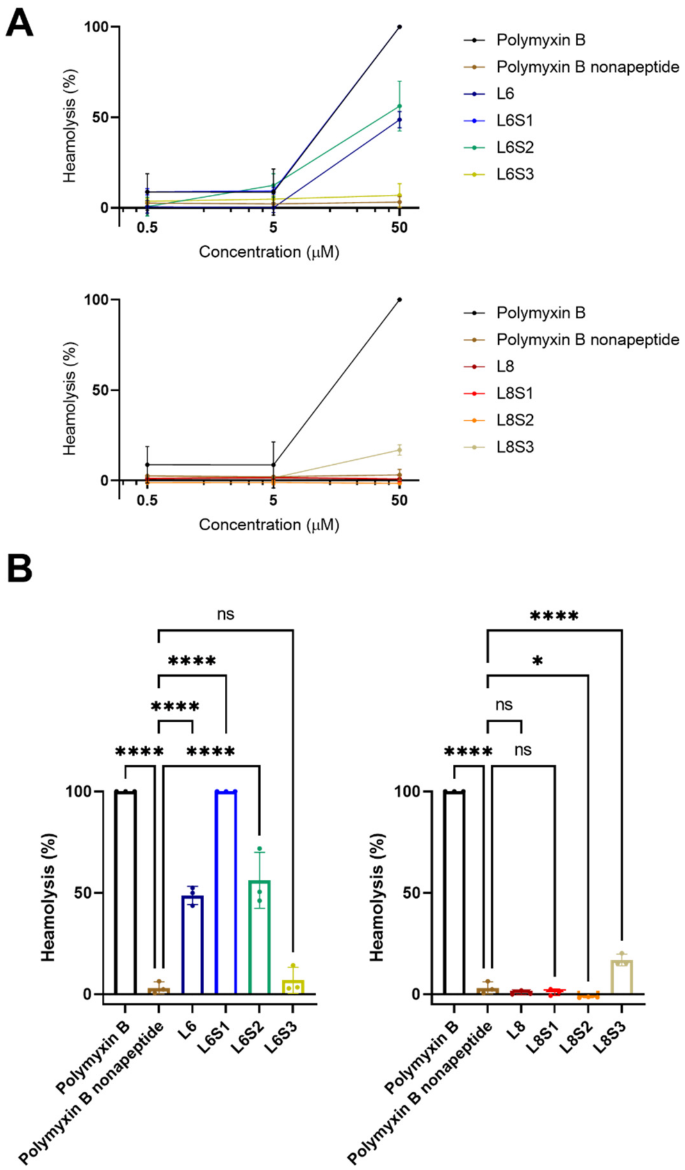

***Supplementary figure S3. Haemolysis of peptide-treated red blood cells**. Sheeps red blood cells were treated with L6 and its variants, L8 and its stapled variants or polymyxin B and polymyxin B nonapeptide for one hour and haemoglobin release was measured (A). Percentage of red blood cell heamolysis was determined as the absorbance of the supernatant of test sample minus the absorbance of supernatant of DMSO treated red blood cells over the absorbance of the supernatant of Triton-X (1%) treated red blood cells minus the absorbance of supernatant of DMSO (1%) treated red blood cells. Percentage of heamolysis of RBCs treated with 50 μM of compound was plotted (B) and statistical significance of the difference of the samples compared to the negative control (polymyxin B nonapeptide) was determined with one-way ANOVA and Dunnett's multiple comparison test (* p<0,05, ** p<0,01, *** p<0,001, **** p<0,0001).*

Peptide design

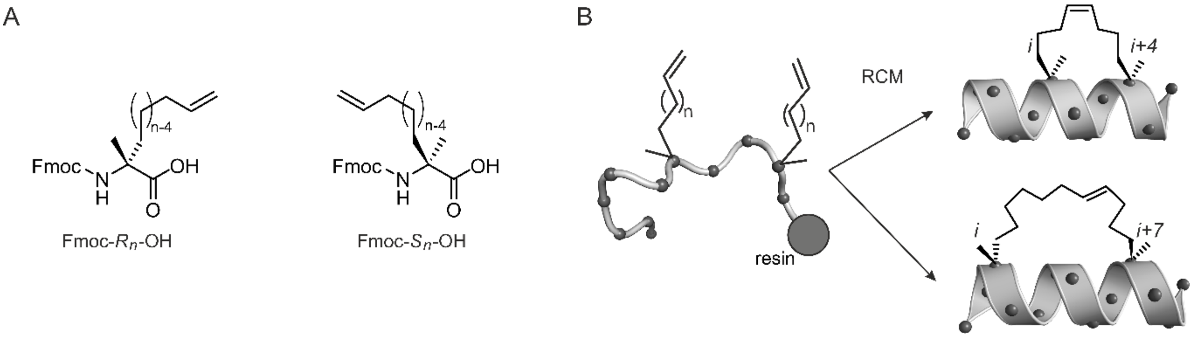

Supplementary figure S4. Design and predicted peptide structures. *Chemical Structure of used unnatural amino acid building blocks (A) and an illustration of the concept of Ring-closing Metathesis and the predicted resulting peptide structures (B).*

Supplementary tables

Peptide analytics

Supplementary table S1 Sequences and analytical details of peptide L6/L8 and stapled variants. *Purity and mass-to-charge ratio was determined via analytical RP-HPLC-MS with absorption at 210 nm. S5: (S)-N-Fmoc-2-(4′-pentenyl)alanine, R8: (R)-N-Fmoc-2-(7′-octenyl)alanine. HPLC chromatograms: 5-95% solvent B (MeCN + 0.1% FA + 0.01% TFA), (H_2O + 0.1% Formic acid (FA) + 0.01% TFA) in 30 min and MS spectrum (positive mode).*

Peptide	Sequence	m/z calc.	m/z found
L6	RRLFRRILRWL	792.5	792.6 $[M+2H]^{2+}$
L6S1	RR(S5)FRR(S5)LRWL	804.0	804.7 $[M+2H]^{2+}$
L6S2	RRLFRR(S5)LRW(S5)	804.0	804.7 $[M+2H]^{2+}$
L6S3	RRL(R8)RRILRW(S5)	808.1	808.7 $[M+2H]^{2+}$
L8	KRIVQRIKKWLR	812.0	812.3 $[M+2H]^{2+}$
L8S1	KR(S5)VQR(S5)KKWLR	824.0	824.1 $[M+2H]^{2+}$
L8S2	KRIVQR(S5)KKW(S5)R	824.0	824.1 $[M+2H]^{2+}$
L8S3	KRI(R8)QRIKKW(S5)R	852.1	852.2 $[M+2H]^{2+}$

In vitro activity of linear and stapled L6 and L8 against Gram-negative bacteria

Supplementary table S2. In vitro antimicrobial activities of L6 and vancomycin against gram-negative clinical isolates. *Indicated are the Minimum inhibitory concentrations (MIC) of L6 alone or in combination with vancomycin, vancomycin alone or in combination with L6 against clinical isolates of E. coli, A. baumannii and K. pneumonia and the respective fractional inhibitory concentration index (*FIC_{index}*) . MIC values were determined as lowest concentration of compound at which 90% of bacterial growth was prevented. The* FIC_{index} *was defined as the sum of the ratios of the MIC value of the peptide in combination with vancomycin and the MIC value of vancomycin in combination with the peptide over MIC value of either the peptide alone or vancomycin alone respectively.*

Clinical isolate	MIC L6 *Mean ± SD (µM)*	MIC L6 + Vancomycin *Mean ± SD (µM)*	MIC Vancomycin *Mean ± SD (µM)*	MIC Vancomycin + L6 *Mean ± SD (µM)*	FIC_{index} *Mean ± SD*
E. coli gsk12	2,14±0,84	8.65±11.53	78.65±5.30	101.08±22.57	2.12±0.41
E. coli 31	3,84±2,48	3.26±2.65	24.52±8.81	24.01±13.39	1.92±0.57
E. coli 87	3,85±1,58	1.83±0.47	40.74±18.17	14.54±5.47	0.98±0.36
A. baumannii 1757	1.1 ± 1.20	0.43 ± 0.31	5.98 ± 4.55	2.68 ± 0.55	1.66 ± 1.03
A. baumannii 2196	0,91±0,59	0.50±0.40	34.67±24	2.24±1.04	0.56±0.33
A. baumannii 1681	0,66±0,13	0.59±0.25	18.98±4.56	23.80±1.71	2.1±0.59
K. pneumoniae 94	0,63±0,29	6.79±10.46	262.54±179.56	260.90±157.53	2.39±0.45
K. pneumoniae 613	0,65±0,15	0.68±0.06	268.87±140.14	172.53±39.46	1.83±0.18
K. pneumoniae 821	2,44±2,17	1.27±0.80	196.82±194.39	173.35±142.32	1.75±0.30

Supplementary table S3. In vitro antimicrobial activities of L8 and vancomycin against gram-negative clinical isolates. *Indicated are the Minimum inhibitory concentrations (MIC) of L8 alone or in combination with vancomycin, vancomycin alone or in combination with L8 against clinical isolates of E. coli, A. baumannii and K. pneumonia and the respective fractional inhibitory concentration index (FIC_{index}). MIC values were determined as lowest concentration of compound at which 90% of bacterial growth was prevented. The FIC_{index} was defined as the sum of the ratios of the MIC value of the peptide in combination with vancomycin and the MIC value of vancomycin in combination with the peptide over MIC value of either the peptide alone or vancomycin alone respectively.*

Clinical isolate	MIC L8<br>*Mean ± SD (µM)*	MIC L8 + Vancomycin<br>*Mean ± SD (µM)*	MIC Vancomycin<br>*Mean ± SD (µM)*	MIC Vancomycin + L8<br>*Mean ± SD (µM)*	FIC_{index}<br>*Mean ± SD*
E. coli gsk12	1,17±0,83	1.54±1.09	78.65±5.3	1.03±0.98	1.32±0.56
E. coli 31	24,56±18,31	6.18±3.79	24.52±8.81	3.29±3.07	0.52±0.05
E. coli 87	26,45±15,09	7.55±4.07	40.74±18.17	4.77±6.13	0.48±0.17
A. baumannii 1757	2.53 ± 1.02	0.92 ± 0.09	5.98 ± 4.55	0.09 ± 0.09	0.40± 0.20
A. baumannii 2196	8,54±5,31	0.33±0.29	34.67±24	0.08±0.14	0.06±0.08
A. baumannii 1681	5,85±0,47	0.48±0.42	18.98±4.56	0.54±0.55	0.11±0.10
K. pneumoniae 94	165,45±1,91	0.95±0.30	262.54±179.56	1.19±1.38	0.02±0.01
K. pneumoniae 613	23,74±29,65	0.66±0.71	268.87±140.14	1.85±1.34	0.05±0.07
K. pneumoniae 821	43,37±28,38	1.57±1.05	196.82±194.39	2.99±3.97	0.05±0.03

Supplementary table S4. In vitro antimicrobial activity of vancomycin, L6 or L8 against A. baumannii in presence of lipopolysaccharide. *Portrayed are the minimum inhibitory concentrations (MIC) of vancomycin, L6 or L8 against A. baumannii 1757 in LB medium without supplement or in LB medium containing soluble smooth LPS (Sm-LPS, 15 µg/ml), soluble rough LPS (Ra-LPS, 15 µg/ml) or soluble deep rough LPS (Rd-LPS, 15 µg/ml). MIC values were determined as lowest concentration of compound at which 90% of bacterial growth was prevented.*

	No supplement<br>Mean ± SD (µM)	+ Sm-LPS (055:B5)<br>Mean ± SD (µM)	+ Ra-LPS (EH100)<br>Mean ± SD (µM)	+ Rd-LPS (F583)<br>Mean ± SD (µM)
MIC Vancomycin	*3.03±2.52*	*5.88±3.56*	*9.86±7.36*	*5.66±4.15*
MIC L6	*1.2±0.43*	*9.85±16.34*	*2.50±3.83*	*0.34±0.40*
MIC L8	*8.78±11.15*	*0.53±0.59*	*11.66±16.07*	*10.83±8.12*

Supplementary table S5. In vitro antimicrobial activities of stapled variants of L6 or L8 and vancomycin against A. baumannii. *Shown are the minimum inhibitory concentrations (MIC) of the indicated compounds, the MIC values of the peptides combined with vancomycin or the MIC values of vancomycin combined with the peptides against A. baumannii 1757 and their respective fractional inhibitory concentration index (FIC_{index}). MIC values were determined as lowest concentration of compound at which 90% of bacterial growth was prevented. The FIC_{index} was defined as the sum of the ratios of the MIC value of the peptide in combination with vancomycin and the MIC value of vancomycin in combination with the peptide over MIC value of either the peptide alone or vancomycin alone respectively.*

Compound	MIC compound *Mean ± SD (µM)*	MIC Peptide + vancomycin *Mean ± SD (µM)*	MIC Vancomycin + peptide *Mean ± SD (µM)*	FIC_{index} *Mean ± SD (µM)*
Vancomycin	5.98 ± 4.55			
L6	1.10 ± 1.20	0.43 ± 0.31	2.68 ± 0.55	1.66 ± 1.03
L6S1	1.80 ± 2.68	1.94 ± 2.80	32.61 ± 24.52	7.38 ± 2.91
L6S2	0.93 ± 0.69	0.64 ± 0.53	1.17 ± 0.54	0.77 ± 0.10
L6S3	1.47 ± 0.48	1.53 ± 0.24	1.70 ± 0.32	1.74 ± 1.03
L8	2.53 ± 1.02	0.92 ± 0.09	0.09 ± 0.09	0.40 ± 0.20
L8S1	12.76 ± 8.91	6.36 ± 6.09	2.84 ± 2.79	0.83 ± 0.63
L8S2	8.69 ± 4.29	0.95 ± 0.51	0.94 ± 0.35	0.14 ± 0.13
L8S3	0.57 ± 0.23	0.57 ± 0.14	3.28 ± 3.69	1.73 ± 1.54

Toxicity of linear and stapled L6 and L8 on Zebrafish larvae

Supplementary table S6. Survival percentages of zebrafish larvae injected with stapled variants of L6 or L8 or combinations of the stapled peptides and vancomycin. *Indicated are the survival percentages of zebrafish larvae injected with indicated compounds or combinations of compounds and Log²FoldChanges compared to vehicle (50% DMSO) injected larvae. Zebrafish larvae were injected with stapled variants of L6 and L8 or combinations of peptide and vancomycin. Surviving larvae were identified by heartbeat over a period of five days post infection. The data are presented as mean survival percentage five days post infection from three independent experiments.*

Injected compound	Survival (%)	Log²FoldChange compared to vehicle injected	Injected compound	Survival (%)	Log²FoldChange compared to vehicle injected
Vehicle	92.63%				
Vancomycin	78,56%	-0.26			
L6	90.00%	-0.04	Vancomycin + L6	100.00%	0.11
L6S1	95.00%	0.04	Vancomycin + L6S1	87.50%	-0.08
L6S2	40.00%	-1.21	Vancomycin + L6S2	70.00%	-0.40
L6S3	70.00%	-0.40	Vancomycin + L6S3	86.67%	-0.10
L8	60.00%	-0.63	Vancomycin + L8	66.67%	-0.47
L8S1	68.00%	-0.45	Vancomycin + L8S1	88.00%	-0.07
L8S2	100.00%	0.11	Vancomycin + L8S2	75.00%	-0.30
L8S3	87.50%	-0.08	Vancomycin + L8S3	95.83%	0.05

In vivo activity of linear and stapled L6 and L8 against *Acinetobacter baumannii*

Supplementary table S7. Survival percentages of A. baumannii infected zebrafish larvae treated with combinations of vancomycin and stapled variants of L6 or vancomycin and stapled variants of L8. *Indicated are the survival percentages of zebrafish larvae injected with indicated compounds or combinations of compounds and Log²FoldChanges compared to vehicle (50% DMSO) injected larvae. Zebrafish larvae were infected with A. baumannii 1757 and injected with indicated treatments. Surviving larvae were identified by heartbeat over a period of five days post infection. The data are presented as mean survival percentage five days post infection from three independent experiments. The fractional inhibitory concentration index (FIC) was defined as the sum of the inverted ratios of the survival percentages of larvae treated with combination treatments and survival percentages of larvae treated with either vancomycin or the peptide. Statistical significance of the difference between combination treatment versus no treatment was determined with the Log-rank (Mantel-Cox) test (* p<0,05, ** p<0,01, *** p<0,001, **** p<0,0001).*

Treatment of infected zebrafish larvae	Survival (%)	Log^2FoldChange compared to vancomycin treatment	FIC_{index}	P-value compared to vancomycin treatment	P-value compared to peptide treatment
**Vehicle**	**13.33%**				
**Vancomycin**	**19,43%**				
L6	4.00%				
**Vancomycin + L6**	**36.00%**	0,89	**0,65**	0,0136 *	0,0011 **
L6S1	4.44%				
Vancomycin + L6S1	2.22%	-3,13	9,74	<0,0001 ****	0,4467 ^{ns}
L6S2	2.22%				
Vancomycin + L6S2	2.22%	-3,13	8,75	<0,0001 ****	0,0002 ***
L6S3	4.44%				
Vancomycin + L6S3	11.11%	-0,81	2,15	0,0527 ^{ns}	0,4314 ^{ns}
L8	20.00%				
Vancomycin + L8	2.50%	-2,96	15,77	< 0,0001 ****	< 0,0001 ****
**L8S1**	**15.00%**				
**Vancomycin + L8S1**	**33.33%**	0,78	**0,85**	0,0045 **	0,0008 ***
L8S2	20.00%				
Vancomycin + L8S2	12.50%	-0,64	3,15	0,6892 ^{ns}	0,5907 ^{ns}
**L8S3**	**36.54%**				
**Vancomycin + L8S3**	**35.85%**	0,88	**1,56**	0,0062 **	0,2418 ^{ns}

Supplementary table S8. Survival percentages of A. baumannii infected zebrafish larvae treated with combinations of rifampicin or erythromycin and L8S1. *Indicated are the survival percentages of zebrafish larvae injected with indicated compounds or combinations of compounds and Log²FoldChanges compared to vehicle (50% DMSO) injected larvae. Zebrafish larvae were infected with A. baumannii and injected with indicated treatments. Surviving larvae were identified by heartbeat over a period of five days post infection. The data are presented as mean survival percentage five days post infection from three independent experiments. The fractional inhibitory concentration index (FIC) was defined as the sum of the inverted ratios of the survival percentages of larvae treated with combination treatments and survival percentages of larvae treated with either vancomycin or the peptide. Statistical significance of the difference between combination treatment versus no treatment was determined with the Log-rank (Mantel-Cox) test (* p<0,05, ** p<0,01, *** p<0,001, **** p<0,0001).*

Treatment of infected zebrafish larvae	Survival (%)	Log²FoldChange compared to antibiotic treatment	FIC_{index}	P-value compared to antibiotic treatment	P-value compared to peptide treatment
Vehicle	13.33%				
Rifampicin	1.67%				
Erythromycin	0.01%				
L8	6.67%				
Rifampicin + L8	2.22%	0,42	3,75	0,7946 ns	0,1342 ns
Erythromycin + L8	0.01%	0,00	>10	0,0012 **	0,0318 *
L8S1	4.00%				
Rifampicin + L8S1	56.67%	5,09	0,1	<0,0001 ****	<0,0001 ****
Erythromycin + L8S1	0.01%	-9,38	>10	0,3034 ns	0,0355 *
